# Postoperative acute kidney injury in adult non-cardiac surgery: joint consensus report of the Acute Disease Quality Initiative and PeriOperative Quality Initiative

**DOI:** 10.1038/s41581-021-00418-2

**Published:** 2021-05-11

**Authors:** John R. Prowle, Lui G. Forni, Max Bell, Michelle S. Chew, Mark Edwards, Morgan E. Grams, Michael P. W. Grocott, Kathleen D. Liu, David McIlroy, Patrick T. Murray, Marlies Ostermann, Alexander Zarbock, Sean M. Bagshaw, Raquel Bartz, Samira Bell, Azra Bihorac, Tong J. Gan, Charles E. Hobson, Michael Joannidis, Jay L. Koyner, Denny Z. H. Levett, Ravindra L. Mehta, Timothy E. Miller, Michael G. Mythen, Mitra K. Nadim, Rupert M. Pearse, Thomas Rimmele, Claudio Ronco, Andrew D. Shaw, John A. Kellum

**Affiliations:** 1 Critical Care and Perioperative Medicine Research Group, William Harvey Research Institute, Queen Mary University of London, London, UK; 2 Intensive Care Unit, Royal Surrey Hospital NHS Foundation Trust, Guildford, UK; 3 Department of Clinical & Experimental Medicine, University of Surrey, Guildford, UK; 4 Department of Physiology and Pharmacology, Karolinska Institutet, Stockholm, Sweden; 5 Department of Anesthesia and Intensive Care, Biomedical and Clinical Sciences, Linköping University Hospital, Linköping, Sweden; 6 Anaesthesia and Critical Care Research Unit, University Hospital Southampton NHS Foundation Trust, Southampton, UK; 7 Division of Nephrology, Department of Medicine, Johns Hopkins University, Baltimore, MD USA; 8 Integrative Physiology and Critical Illness Group, Clinical and Experimental Sciences, Faculty of Medicine, University of Southampton, Southampton, UK; 9 Divisions of Nephrology and Critical Care, Departments of Medicine and Anesthesia, University of California San Francisco School of Medicine, San Francisco, CA USA; 10 Department of Anesthesiology, Vanderbilt University Medical Center, Nashville, TN USA; 11 School of Medicine, University College Dublin, Dublin, Ireland; 12 Department of Intensive Care, Guy’s & St Thomas’ NHS Foundation Hospital, London, UK; 13 Department of Anesthesiology, Intensive Care and Pain Medicine, University Hospital Münster, Münster, Germany; 14 Department of Critical Care Medicine, Faculty of Medicine and Dentistry, University of Alberta and Alberta Health Services, Edmonton, AB Canada; 15 Division of Critical Care Medicine, Department of Anesthesia, Duke University Medical Center, Durham, NC USA; 16 Division of Population Health and Genomics, University of Dundee, Dundee, Scotland UK; 17 Precision and Intelligent Systems in Medicine (PrismaP), Division of Nephrology, Hypertension, and Renal Transplantation, University of Florida, Gainesville, FL USA; 18 Department of Anesthesiology, Stony Brook University Renaissance School of Medicine, Stony Brook, NY USA; 19 The PACES Center for Value in Healthcare, Framingham, MA USA; 20 Division of Intensive Care and Emergency Medicine, Department of Internal Medicine, Medical University Innsbruck, Innsbruck, Austria; 21 Section of Nephrology, Department of Medicine, University of Chicago, Chicago, IL USA; 22 Division of Nephrology, Department of Medicine, University of California, San Diego, CA USA; 23 Department of Anesthesiology, Duke University School of Medicine, Durham, NC USA; 24 University College London Hospitals NIHR Biomedical Research Centre, London, UK; 25 Division of Nephrology and Hypertension, Department of Medicine, Keck School of Medicine, University of Southern California, Los Angeles, CA USA; 26 Department of Anesthesiology and Intensive Care Medicine, Edouard Herriot Hospital, Hospices Civils de Lyon, Lyon, France; 27 Department of Nephrology, Dialysis and Transplantation, San Bortolo Hospital, International Renal Research Institute of Vicenza, and Department of Medicine, University of Padova, Padova, Italy; 28 Department of Anesthesiology and Pain Medicine, Alberta Health Services, Edmonton, AB Canada; 29 Center for Critical Care Nephrology, Department of Critical Care Medicine, University of Pittsburgh, Pittsburgh, PA USA

**Keywords:** Kidney, Acute kidney injury, Surgery

## Abstract

Postoperative acute kidney injury (PO-AKI) is a common complication of major surgery that is strongly associated with short-term surgical complications and long-term adverse outcomes, including increased risk of chronic kidney disease, cardiovascular events and death. Risk factors for PO-AKI include older age and comorbid diseases such as chronic kidney disease and diabetes mellitus. PO-AKI is best defined as AKI occurring within 7 days of an operative intervention using the Kidney Disease Improving Global Outcomes (KDIGO) definition of AKI; however, additional prognostic information may be gained from detailed clinical assessment and other diagnostic investigations in the form of a focused kidney health assessment (KHA). Prevention of PO-AKI is largely based on identification of high baseline risk, monitoring and reduction of nephrotoxic insults, whereas treatment involves the application of a bundle of interventions to avoid secondary kidney injury and mitigate the severity of AKI. As PO-AKI is strongly associated with long-term adverse outcomes, some form of follow-up KHA is essential; however, the form and location of this will be dictated by the nature and severity of the AKI. In this Consensus Statement, we provide graded recommendations for AKI after non-cardiac surgery and highlight priorities for future research.

## Abstract (web summary)

The development of acute kidney injury (AKI) after major non-cardiac surgery is associated with substantial long-term morbidity and mortality. This joint Consensus Statement from the Acute Disease Quality Initiative and the PeriOperative Quality Initiative provides recommendations for the definition, prevention and management of postoperative AKI.

## Introduction

The development of postoperative complications after major non-cardiac surgery — particularly acute kidney injury (AKI) — has consistently been associated with substantial long-term morbidity and mortality^1–4^. AKI can be considered a sentinel postoperative complication that is strongly associated with increased risk of death, co-occurrence of other postoperative complications, increased length of hospital stay and the development of progressive chronic kidney disease (CKD), which results in a considerable health-care and societal burden^5–7^.

Considerable research and previous consensus meetings have focused on AKI after cardiac surgery^8^. However, the role of AKI after other forms of major surgery, particularly gastrointestinal surgery, has received less attention, even though such procedures are associated with higher 30-day mortality than many forms of cardiac surgery^9^. Despite the importance of AKI as a complication after all forms of major surgery, lack of consensus exists with regard to the definition, prevention and treatment of postoperative AKI (PO-AKI) and the pathophysiology of PO-AKI in the non-cardiac setting has not been well described. Furthermore, the understanding of and approach to this condition can differ between specialists in nephrology, anaesthesia and/or perioperative medicine.

In 2019, a joint meeting of the Acute Disease Quality Initiative (ADQI-24) and the PeriOperative Quality Initiative (POQI-7) was convened to address PO-AKI after major non-cardiac surgery. Here, we present our recommendations for clinical practice as well as for further research in this important area.

## Methods

The Conference Chairs of the 24th ADQI/7th POQI consensus committee (J.R.P., J.A.K., A.D.S. and L.G.F.) convened a diverse panel of clinicians and researchers representing relevant disciplines (internal medicine, nephrology, critical care, anaesthesia, perioperative medicine, pharmacy and surgery) from Europe, North America and Australia, to discuss the issues relating to PO-AKI. The consensus meeting was held at Emmanuel College, Cambridge, UK, on 4–7 September 2019, and followed the established ADQI process and POQI protocols, including use of a modified Delphi method to achieve consensus^8,10^. The broad objective of ADQI 24 was to produce expert-based statements and a summary of current knowledge pertaining to the definition and management of PO-AKI in the setting of non-cardiac surgery for use by clinicians and researchers, based on review of evidence by and professional judgment of the faculty as well as to identify evidence gaps to establish research priorities.

Conference participants were divided into five work groups: group 1 was tasked with the epidemiology and pathophysiology of PO-AKI. Group 2 was tasked with developing a definition for PO-AKI. Group 3 developed recommendations for the prevention of PO-AKI. Group 4 focused on treatment options for patients with PO-AKI, and group 5 explored outcomes after PO-AKI (Supplementary Information). Members of the work groups performed comprehensive literature searches and developed a consensus of opinion, backed by evidence where possible, to distil the available literature and articulate a research agenda to address important unanswered questions. Members assessed the level of evidence and strength of recommendation for all consensus statements using the GRADE evidence to decision frameworks^11^. A number of ungraded research recommendations were also identified in areas where high-quality evidence is lacking. Strength of recommendations and aggregated conclusions were established by consensus of all of the participants, who formally voted and approved the consensus recommendations.

## Pathophysiology and epidemiology

**Fig. 1:**
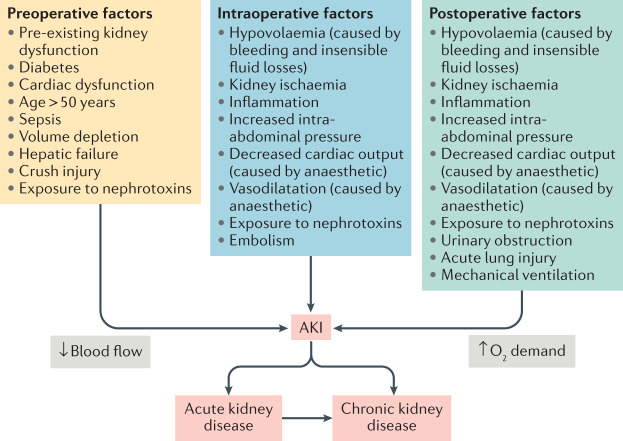
Pathophysiology of PO-AKI. Similar to most other forms of acute kidney injury (AKI), postoperative AKI (PO-AKI) commonly has a multifactorial aetiology, which is mediated by common injury pathways that affect the kidney microcirculation, oxygen demand and inflammation. In most cases, a combination of preoperative risk factors, intraoperative events and postoperative events leads to the development of AKI. Baseline risk factors and the persistence and severity of injurious factors in the postoperative setting also determine the outcomes of AKI, acute kidney disease and eventually chronic kidney disease. Adapted from Acute Disease Quality Initiative 24, www.ADQI.org, CC BY 2.0 (https://creativecommons.org/licenses/by/2.0/).

Like all forms of AKI, PO-AKI represents a clinical syndrome rather than a single disease and limited histological data from kidney biopsy samples are available to identify specific pathology^12,13^ (Box 1). Animal models that are used to study AKI pathophysiology tend to employ a single insult to the kidneys rather than explore the multifactorial insults that are common in clinical practice^14–18^. However, commonly implicated mechanisms for PO-AKI include ischaemia–reperfusion injury, endogenous or exogenous nephrotoxins, physical causes such as obstruction, inflammatory factors, vasoconstriction, and oxidative stress^19^ (Fig. 1). Thus, a variety of exposures that are encountered in the surgical setting are involved in the development of PO-AKI^20^. Many of these exposures might also have a role in other forms of AKI, but their relative importance, severity and timing in PO-AKI may differ from that of other AKI settings. Within this framework, the contribution of individual mechanisms is often difficult to determine; for instance, nephrotoxic drugs have been implicated in 20–30% of all AKI episodes and are commonly encountered in the surgical setting^21^. Moreover, agents such as radiocontrast and antibiotics are commonly used in patients who are already at risk of AKI and discerning their relative contribution to the overall AKI course is often difficult. Future research including studies aimed at understanding the interplay between concomitant perioperative complications might help to identify the underlying pathophysiological mechanisms of PO-AKI.

Box 1 Pathophysiology and epidemiology of PO-AKI **Consensus Statement 1a**

Postoperative acute kidney injury (PO-AKI) is a clinical syndrome rather than a single disease. The majority of cases of PO-AKI are multifactorial in cause (ungraded).

**Consensus Statement 1b**

The incidence of PO-AKI (defined by changes in serum creatinine) varies with the characteristics and urgency of surgery. The incidence of AKI after day or ambulatory surgery is uncertain (ungraded).

**Consensus Statement 1c**

AKI defined by transient oliguria is more common in the intraoperative and postoperative period than AKI defined by serum creatinine. Severe oliguria and anuria, even in the absence of changes in serum creatinine, are associated with increased long-term morbidity and mortality (ungraded).

**Consensus Statement 1d**

The majority of observational studies focus on AKI in the immediate postoperative period. Little evidence is available regarding the epidemiology of acute kidney disease in the perioperative setting (ungraded).

**Consensus Statement 1e**

Risk factors for PO-AKI include age >50 years, male sex, glomerular filtration rate <60 ml/min/1.73 m^2^, diabetes mellitus, heart failure, ascites, hypertension, emergency surgery, intraperitoneal surgery, number of medications, use of angiotensin-converting enzyme (ACE) inhibitors or angiotensin receptor blockers (ARBs), high American Society of Anesthesiology Physical Status classification score and albuminuria. Patients with chronic kidney disease (CKD) and/or diabetes are at a particularly high risk of AKI (ungraded).

### Epidemiology of postoperative AKI

A variety of criteria for changes in serum creatinine levels and urine output have been used to define AKI; however, the perioperative medicine community has largely adopted the 2012 Kidney Disease: Improving Global Outcomes (KDIGO) consensus criteria to define PO-AKI in clinical trials and epidemiological studies^22^. Adoption of this consensus definition has resulted in a greater appreciation of the incidence, varied presentation and outcomes of PO-AKI (Box 1). Although changes in serum creatinine are fairly common in the postoperative period, the incidence of PO-AKI varies according to the characteristics and urgency of surgery as well as the exact AKI definition employed, with a higher incidence in emergency surgery^2,4,23–31^ (Supplementary Table 1). The incidence of AKI in day or ambulatory surgery is unknown — but presumed to be low — owing to a lack of published data on the detection of AKI among patients who are discharged to the community immediately following surgery.

More is known about PO-AKI defined by change in serum creatinine than that defined by perioperative decrements in urine output. The prognostic implications of intraoperative transient oliguria are controversial, with some studies reporting a higher risk of subsequent adverse outcomes and others suggesting no increase in risk^32–35^. One study suggested that discrimination for subsequent adverse outcomes could be enhanced by testing for novel biomarkers. In this study, which included some patients with PO-AKI, the sum of the concentration of tissue inhibitor of metalloproteinases 2 (TIMP2) multiplied by that of insulin-like growth factor binding protein 7 (IGFBP7) added prognostic information to AKI defined by urine output alone ([TIMP2]×[IGFBP7] > 2 was associated with progression from stage 1 AKI)^36^.

Many, but not all, of the available studies suggest that PO-AKI defined by oliguria alone is more common than PO-AKI defined by changes in serum creatinine levels^26,37–39^. In a study of PO-AKI after non-cardiac surgery, the incidence of AKI increased from 8% to 64% if urine output was included in the definition^37^. Some postoperative oliguria is assumed to be an appropriate physiological response to decreased intravascular volume, vasodilatation or non-osmotic release of arginine vasopressin in response to tissue injury^40^; however, sustained decrements in urine output, even without accompanying changes in serum creatinine, have been associated with longer-term morbidity and mortality^38,41^.

## Epidemiology of postoperative AKD

The concept of acute kidney disease (AKD) is intended to bridge the gap between AKI, which refers to an acute alteration in kidney function over a 7-day period, and CKD, which refers to a sustained alteration in kidney function over a 3-month period. AKD therefore refers to AKI that persists for more than 7 days after the initial insult, or a progressive decline in kidney function that does not meet AKI criteria on a week-by-week basis. Few epidemiological data are available on the incidence and prognosis of postoperative AKD, despite its likely importance as a bridge to development of sustained kidney dysfunction and CKD^42^. A study that evaluated substantial decline in kidney function, defined as a 30% decrease in estimated glomerular filtration rate at 60 days after surgery, suggested that the incidence of this outcome was only 2% among patients without PO-AKI (defined by change in serum creatinine only), but 10%, 17% and 29% among patients who experienced PO-AKI stages 1, 2, and 3, respectively^43^. The incidence of AKD in the general population is likely lower than in the post-surgery population; in a study of Canadian electronic health records, 5.2% of patients in a given period had AKD with or without AKI^44^.

### Risk factors for postoperative AKI

Several patient-related, surgery-related, and anaesthetic-related risk factors for developing early PO-AKI during the first 48 h after surgery have been described^4,40^. The three validated risk scores for PO-AKI include male sex, age >50 years, diabetes mellitus, hypertension, ascites, heart failure, emergent surgery, intraperitoneal surgery, poly-pharmacy, use of an angiotensin-converting enzyme (ACE) inhibitor or angiotensin receptor blocker (ARB), and increasing American Society of Anesthesiologists physical status classification score^34,45,46^. Albuminuria and hypoalbuminaemia have also been described as risk factors for PO-AKI^47–50^. Risk factors for PO-AKI occurring in patients in the ICU more than 48 h after surgery reflect risk factors for AKI in critical illness including the need for new nephrotoxic medication, sepsis and shock^51,52^. Whether targeting modifiable risk factors for early PO-AKI, for example, using perioperative haemodynamic interventions, will impact the incidence of AKI later in the postoperative period, >48 h after surgery, remains uncertain.

Use of a validated clinical risk score is a potentially useful approach to risk stratify patients for targeted interventions or to facilitate the conduct of randomized controlled trials (RCTs)^53^. However, existing validated risk scores for PO-AKI were developed using a variety of older (pre-KDIGO) definitions of AKI or were applied to select surgical settings^34,45,46^. More generalizable prognostic clinical tools are needed to accurately risk-stratify patients preoperatively, particularly to distinguish those patients at the highest levels of risk (e.g. >20–30%). In the absence of such tools, patients with CKD and those with diabetes can reasonably be considered to be at increased risk of PO-AKI. The KDIGO guideline for the diagnosis, evaluation and management of AKI suggests that age >60 years, emergency surgery, elevated American Society of Anesthesiologists physical status classification score and preoperative comorbid illnesses, including diabetes and any chronic disease of the heart, lung or liver, are risk factors for PO-AKI^42^.

### Research recommendations

Understanding the pathophysiology and epidemiology of PO-AKI is central to the identification and development of novel preventative therapies and treatments. This understanding may be improved by conducting prospective studies that include collection of biological samples for histological and biomarker examination as well as retrospective analyses that examine shared pathogenesis across a spectrum of perioperative complications. The incidence of PO-AKI in the day and ambulatory surgery settings is uncertain and should be investigated in epidemiological studies. Development of better risk prediction tools for PO-AKI could help to risk-stratify patients and set thresholds of predicted risk that merit adjustments in perioperative care.

## Definition of postoperative AKI

Although the KDIGO definition of AKI is now widely employed in the perioperative literature, the exact implementation of this definition varies considerably in terms of timeframe of diagnosis. We recommend defining PO-AKI as occurring when existing KDIGO criteria for AKI are met within 7 days of an operative intervention (Box 2). AKI occurring de novo ≥7 days after surgery may arise in a variety of contexts that are not necessarily related to the surgery itself; we recommend that such AKI should be evaluated and managed as hospital-acquired AKI. Our recommendation for using a 7-day window is somewhat arbitrary but maintains important consistency with the KDIGO definitions for AKI in other clinical contexts^54^ and is supported by evidence from numerous clinical studies^51,55–57^. Furthermore, within the 7-day window, different timing of AKI may be indicative of the nature of the kidney insult, that is, intra-operative events versus post-operative insults.

Notably, AKI could potentially be present before surgery (for example, in patients with sepsis requiring surgical intervention, acute trauma or preoperative contrast exposure), highlighting the importance of a risk-based kidney health assessment (KHA)^51^. Use of a KHA was previously recommended by ADQI in the context of quality improvement processes for monitoring patients at a high risk of AKI before surgery^58^. A KHA comprises a structured evaluation that includes previous history of AKI, current medications, cardiovascular health, haemodynamic status and markers of kidney function (i.e. serum creatinine) and kidney damage (i.e. urine dipstick). Use of a KHA in the perioperative period involves integrated assessment of patient-specific and procedure-specific risk factors, clinical context and resource setting.

**Fig. 2:**
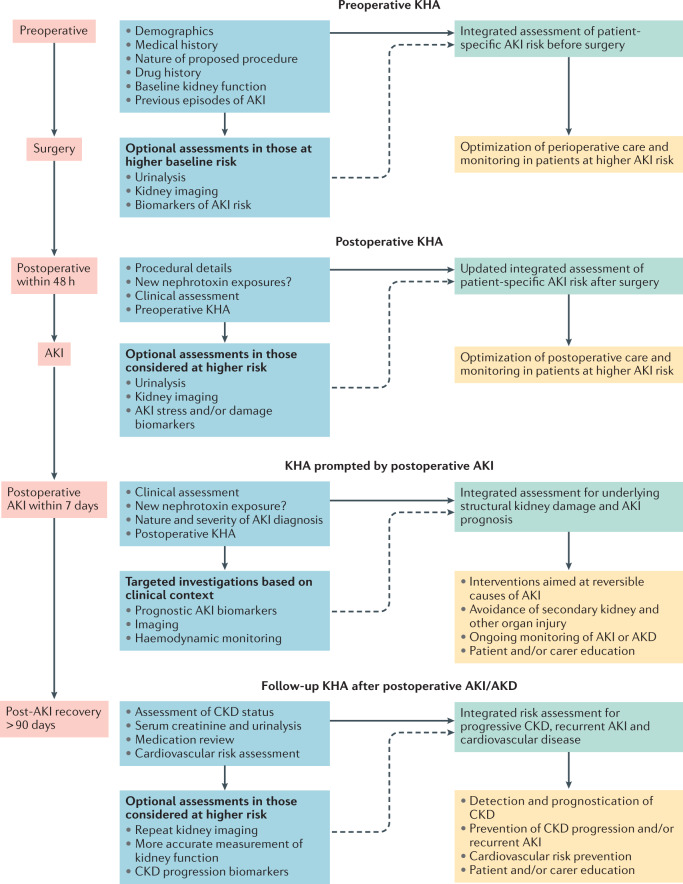
The role of the KHA in postoperative AKI. Kidney health assessments (KHAs) can be used in the risk assessment, detection, management and follow-up of postoperative acute kidney injury (AKI). A series of context-specific KHAs involving integration of medical history and clinical context, potentially in combination with further investigations, such as analysis of specific kidney biomarkers or imaging, in higher-risk settings, can provide kidney prognostic information to guide further monitoring and treatment. AKD, acute kidney disease; CKD, chronic kidney disease. Adapted from Acute Disease Quality Initiative 24, www.ADQI.org, CC BY 2.0 (https://creativecommons.org/licenses/by/2.0/).

Importantly, the KHA approach can be used to perform context-dependent evaluation both before and after surgery, when AKI is diagnosed and during follow-up (Fig. 2). A perioperative KHA may range from a simple history and physical examination conducted as part of a routine low-risk preoperative evaluation, to an evaluation that includes more specific measures such as urinalysis to identify proteinuria, biomarkers of kidney damage and kidney imaging in higher-risk patients (Box 2). Measurements other than serum creatinine and urine output are not currently part of the KDIGO AKI definition, but likely provide important context for enhanced interpretation of these criteria. Although we strongly support routine use of KHAs before and after surgery, the potential clinical benefit of this approach has not been formally evaluated and so remains uncertain. However, an accepted advantage of routine perioperative KHAs is improved recognition of preoperative kidney dysfunction, which establishes a baseline for subsequent diagnosis of PO-AKI.

Conventional diagnostic criteria for AKI might be influenced by multiple factors specific to the perioperative period, rendering their predictive utility for PO-AKI less certain. Serum creatinine concentration is subject to abrupt changes in total body water^59^, which may spuriously increase or decrease measured values, as well as perioperative changes in creatinine production that alter serum creatinine levels independently of changes in GFR^60^. Urine output is also likely to be influenced by multiple physiological factors specific to the perioperative period, including intravascular volume status, relative or absolute hypotension and neurohormonal response to surgery. Data suggest that although intraoperative oliguria is associated with PO-AKI, the positive predictive value is low^32^. We therefore recommend that urine output is interpreted in the context of severity of oliguria, clinical setting and other corroborating evidence of PO-AKI. Thus, isolated perioperative oliguria meeting AKI stage 1 criteria in the context of transient hypotension should not be viewed as being the same as more severe or prolonged oligo-anuria unresponsive to haemodynamic interventions. The presence of new evidence of kidney tubular damage (e.g. on urine microscopy or from novel biomarkers) or evidence of evolving sepsis in the context of early oliguria would be suggestive of AKI; these factors can be identified and integrated within a timely postoperative KHA.

Box 2 Definition of PO-AKI **Consensus Statement 1a**

We recommend defining postoperative acute kidney injury (PO-AKI) as occurring when existing Kidney Disease Improving Global Outcomes (KDIGO) criteria for AKI are met within 7 days of an operative intervention (ungraded).

**Consensus Statement 1b**

We recommend performing a risk-based Kidney Health Assessment (KHA) preoperatively and postoperatively, with the specific methods and frequency of assessment determined by clinical judgement based on individual patient-specific and procedural-specific risk factors and the available resources **(grade D evidence; strong recommendation)**.

**Consensus Statement 1c**

We recommend using the term postoperative acute kidney disease (PO-AKD) when KDIGO criteria for AKI are present after postoperative day 7 and objective evidence of injury initiation was present within the first 7 postoperative days (ungraded).

### Definition of postoperative AKD

**Fig. 3:**
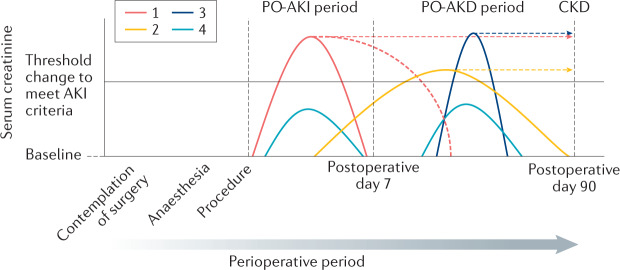
Conceptual model of PO-AKI and PO-AKD. Postoperative acute kidney injury (PO-AKI) occurs when the Kidney Disease Improving Global Outcomes (KDIGO) criteria for AKI are met within 7 days of an operative intervention. Postoperative acute kidney disease (PO-AKD) occurs when patients with PO-AKI continue to meet KDIGO AKI criteria ≥7 days after surgery or when patients whose serum creatinine levels began to rise following surgery meet KDIGO AKI criteria ≥7 days after surgery. A number of potential trajectories of serum creatinine are depicted with suggested application of the proposed nomenclature. PO-AKI might commence and resolve before postoperative day 7 or persist after postoperative day 7 and therefore be classed as PO-AKD. If PO-AKD continues after postoperative day 90 it will be classed as chronic kidney disease (CKD) (trajectory 1). PO-AKD also occurs when evidence of new kidney injury was present before postoperative day 7 but did not meet the criteria for PO-AKI until after postoperative day 7. This form of PO-AKD might also either recover before postoperative day 90 or continue after postoperative day 90 and be classed as CKD (trajectory 2). Stand-alone AKI or AKD (that is, AKI or AKD that is seemingly not related to the operative intervention) can also occur during the perioperative period. As these new events occur distant to the surgical insult they should not be referred to as PO-AKI or PO-AKD and should be considered in the context of their direct precipitants (trajectory 3). Subclinical kidney injury can occur before or after postoperative day 7 (trajectory 4). This subclinical injury does not meet current criteria for AKI or AKD, but may be identified by risk-based serial kidney health assessments (KHAs). Source: Adapted from Acute Disease Quality Initiative 24, www.ADQI.org, CC BY 2.0 (https://creativecommons.org/licenses/by/2.0/).

Consistent with the definition of AKD as a condition in which AKI stage 1 or greater is present ≥7 days after an AKI initiating event, postoperative acute kidney disease (PO-AKD) occurs in patients with PO-AKI who continue to meet KDIGO criteria for AKI >7 days after surgery. Patients whose rise in serum creatinine level begins before postoperative day 7 but whose serum creatinine level does not increase ≥50% above baseline until 7–90 days after surgery would also meet PO-AKD criteria based on a slow progressive deterioration in kidney function in the postoperative period (Box 2). However, the greater the time interval between surgery and identification of kidney injury the less likely that the injury could be attributed to the perioperative process. Patients who meet criteria for AKI for the first time after postoperative day 7 without any previous increase in serum creatinine should therefore be referred to as having AKI^54^ or AKD^61^ without the postoperative prefix. With this proposed nomenclature (Fig. 3), it sought to combine a level of consistency with existing recommendations for use of AKI and AKD terminology^54,61^. However, revision may be warranted as our understanding of the pathophysiology and prognostic implications of temporal variations in the postoperative disease course evolves.

### Research recommendations

Limited information exists regarding the prognostic implications of the nature and timing of the AKI diagnosis within 7 days after surgery, and these important aspects of the PO-AKI diagnostic criteria warrant better characterization. Furthermore, to what extent indices of kidney injury other than serum creatinine and urine output, such as urinary or plasma biomarkers, are important for prognostication in PO-AKI is unknown and should be the subject of further study. Whether or not important differences exist in the epidemiology and prognostic significance of PO-AKD that arises as persistent PO-AKI compared with PO-AKD that develops after a gradual rise in creatinine after day 7 is also unknown.

The impact of routine, structured KHA on the detection of PO-AKI remains to be determined. An expanded perioperative urinalysis conducted as part of a detailed KHA might identify previously unknown pathology and could potentially lead to additional, potentially expensive and/or invasive investigations that could delay surgery. The presumed benefits and cost-effectiveness of a KHA therefore require confirmation. Finally, the optimal interpretation of intraoperative and postoperative oliguria remains unclear and should be the subject of further study.

## Prevention of postoperative AKI

### Preoperative strategies

Surgery is sometimes performed in patients with established AKI; however, common perioperative interventions focus on prevention of PO-AKI (Box 3). As nephrotoxic drugs are an important preventable contributor to the risk of PO-AKI, exposure to these drugs should be limited whenever possible and the benefits of the intervention weighed against the risk of developing or worsening AKI^62^. Although the avoidance of potential nephrotoxins is considered to be a cornerstone of AKI prevention, there is a relative paucity of data supporting this approach. However, routine use of gentamicin for surgical prophylaxis was associated with an increased risk of AKI following orthopaedic surgery^63^ and, in the paediatric setting, systematic screening for nephrotoxin use, with a focus on close monitoring in patients exposed to multiple nephrotoxins and nephrotoxin cessation, has been shown to reduce rates of AKI^64,65^.

At present, common practice is to discontinue use of ACE inhibitors and ARBs before surgery to avoid perioperative hypotension. This approach is assumed to reduce the risk of perioperative AKI based on physiological rationale. In line with this established practice, we recommend discontinuing ACE inhibitors and ARBs for a minimum of 24 h before surgery to minimize the risks of perioperative hypotension and/or postoperative AKI (Box 3). This recommendation is weak owing to limited data in support of this practice and is likely to be revised as more evidence becomes available. For instance, an analysis of 949 patients undergoing major gastrointestinal or hepatobiliary surgery failed to show a difference in the rates of AKI between those who did and those who did not have ACE inhibitors and ARBs withheld before surgery^66^.

NSAIDs are generally avoided in surgical patients at a high risk of AKI, but might be beneficial as opiate-sparing agents in patients who are at a low risk of AKI. Although NSAIDs are widely used in patients with normal kidney function, their impact on risk of AKI in the postoperative period is unclear^24^.

To date, no preoperative pharmacological intervention has been shown to reduce the risk of PO-AKI in RCTs. However, attention should be paid to ensuring euvolaemia prior to surgery and addressing or avoiding preoperative fluid losses caused by excessive fasting, bowel preparation and/or acute illness^67^.

Box 3 Prevention of PO-AKI: preoperative strategies **Consensus Statement 3a**

We recommend that when considering use of medications or interventions that are associated with an increased risk of acute kidney injury (AKI), the relative benefits and/or necessity of the medications or interventions should be balanced with the risk of postoperative AKI (PO-AKI). This recommendation applies to outpatient medications, new medications and exposures including contrast media **(grade C evidence; strong recommendation)**.

**Consensus Statement 3b**

We recommend discontinuing angiotensin-converting enzyme (ACE) inhibitors and angiotensin receptor blockers (ARBs) for a minimum of 24 h before surgery, dependent on the specific medication, to minimize the risks of perioperative hypotension and/or PO-AKI **(grade D evidence, weak recommendation)**.

**Consensus Statement 3c**

We recommend assessing the relative risks and benefits of use of NSAIDs (including COX2 inhibitors) in individual patients. In general, in patients at a high risk of PO-AKI, the risk of AKI associated with use of NSAIDs might outweigh the benefits with regard to opioid minimization, whereas in low-risk patients, the benefits of use of NSAIDs might outweigh the risks. The risk–benefit profile in patients at a moderate risk of AKI is particularly uncertain **(grade D evidence; strong recommendation)**.

### Intraoperative strategies

Intravenous fluid and haemodynamic management in the intraoperative and early postoperative period has a major impact on the development of PO-AKI (Box 4). The RELIEF RCT in 3,000 patients undergoing major elective non-cardiac surgery compared a zero fluid balance target with a “moderately liberal” approach to fluid administration and demonstrated an increased risk of AKI in the restrictive, zero fluid balance group^68^. Algorithms that use cardiac output monitoring to optimize cardiac stroke volume and/or increase global oxygen delivery, known as goal-directed therapy, have been the subject of multiple RCTs. A Cochrane review and meta-analysis suggested that goal-directed therapy can reduce the risk of PO-AKI^69,70^. Large observational studies have shown strong associations between intraoperative hypotension and PO-AKI with the risk of organ injury a function of the severity and duration of the hypotension^71–73^. Both absolute mean arterial blood pressure (MAP (e.g. 60–70 mmHg)) and relative MAP (e.g. <30% reduction from baseline) thresholds are associated with PO-AKI. The results of an interventional trial of use of a MAP target of >65 mmHg as part of a goal-directed strategy have added weight to these observational data, demonstrating fewer postoperative complications (including AKI) and shorter length of hospital stay with this approach^74^. Similar to other settings such as sepsis, a higher MAP target may be beneficial in reducing the incidence of AKI in patients with poorly controlled pre-existing hypertension^75^.

Fluid composition may also affect the risk of PO-AKI; 0.9% saline is associated with increased risk of PO-AKI compared with balanced crystalloids^76–78^ (Box 4). High-quality evidence has shown that use of starch-based colloids increases the risk of AKI and other adverse outcomes in patients with sepsis and other critically ill patients^79,80^. Although caution is advised when using starch-based colloids, the limited data in the elective perioperative setting, where lower fluid volumes may be given than in treatment of septic shock, suggest little difference in kidney outcomes when starch-based colloids are used in preference to crystalloids or non-starch colloids^81,82^. Ongoing research is investigating use of starch-based colloids versus crystalloids in the perioperative setting. One such trial published in 2020 reported no significant difference in a composite outcome of death or postoperative complications within 14 days of major abdominal surgery between patients who received low-molecular-weight hydroxyethyl starch compared with those who received 0.9% saline^83^.

Box 4 Prevention of PO-AKI: intraoperative strategies **Consensus Statement 4a**

We recommend not using restrictive or zero-balance perioperative fluid regimens in major elective surgery (except in specific circumstances). Such regimens are associated with increased risk of postoperative acute kidney injury (PO-AKI) **(grade B evidence; strong recommendation)**.

**Consensus Statement 4b**

We recommend the use of goal-directed haemodynamic therapy in high-risk patients to optimize volume status, blood pressure and cardiac output and reduce the risk of PO-AKI **(grade B evidence; strong recommendation)**.

**Consensus Statement 4c**

We recommend maintaining an intraoperative mean arterial blood pressure (MAP) >65 mmHg to reduce the risk of PO-AKI. The risk of AKI is a function of both the magnitude of hypotension and its duration. For selected patients, including those with pre-existing systemic hypertension, a higher MAP target should be considered **(grade C evidence; weak recommendation)**.

**Consensus Statement 4d**

We recommend the perioperative use of balanced crystalloids rather than 0.9% saline to reduce the risk of PO-AKI **(grade C evidence; strong recommendation)**.

**Consensus Statement 4e**

In the setting of elective surgery outside of the ICU, insufficient evidence exists to make a recommendation regarding the choice of crystalloids or colloids for volume expansion to modify the risk of PO-AKI (ungraded).

### Postoperative strategies

The early postoperative period has not been extensively studied in relation to kidney outcomes. However, therapeutic strategies used in the hours after surgery could likely modify the risk of AKI (Box 5). Clinically important postoperative hypotension (defined as systolic blood pressure <90 mmHg and requiring medical intervention) affects over 30% of patients, often goes uncorrected for longer than intraoperative hypotension and is associated with an increased risk of all-cause mortality and myocardial injury^84^. These findings suggest that maintenance of fluid and haemodynamic status in the early postoperative phase is important to avoid organ injuries including AKI^28^. A trial that used stroke volume optimization and vasopressors to target a MAP within 10% of the preoperative baseline value during and for 4 h after surgery showed that this strategy was associated with a reduction in postoperative organ injury, but was not powered to assess kidney-specific outcomes^85^. Further research is warranted to evaluate postoperative monitoring strategies and ideal clinical settings to reduce the risk of PO-AKI in moderate- to high-risk patients.

As postoperative hyperglycaemia is strongly associated with AKI, avoidance of perioperative hyperglycaemia (>180 mg/dl) is recommended^86^. Failure to restart chronic ACE inhibitor or ARB therapy that was suspended preoperatively is associated with increased 30-day mortality^87,88^. However, the optimal time point to restart these medications has not been clearly established. Given their effects on the kidney, a reasonable approach would be to conduct a focused KHA before restarting ACE inhibitors or ARBs.

Enhanced recovery after surgery pathways are typically multimodal, multidisciplinary interventions that have been promoted to facilitate recovery from surgery. Use of NSAIDs to minimize opiate analgesia requirements is a common component of such pathways, along with avoidance of markedly positive fluid balance. Accordingly, in some settings such as colorectal surgery, implementation of an enhanced recovery after surgery pathway could be associated with a higher, rather than lower, incidence of AKI^89^.

Box 5 Prevention of PO-AKI: postoperative strategies **Consensus Statement 5a**

We recommend maintenance of adequate organ perfusion, including attention to euvolaemia and treatment of hypotension in the early postoperative period, to reduce the risk of postoperative acute kidney injury (PO-AKI). Monitoring and targeting haemodynamic parameters may require enhanced levels of monitoring and/or care depending on the clinical context and patient risk profile **(grade D evidence; strong recommendation)**.

**Consensus Statement 5b**

We recommend treatment of postoperative hyperglycaemia (target glucose <180 mg/dl [<10 mmol/l]) to reduce the risk of PO-AKI **(grade C evidence; strong recommendation)**.

**Consensus Statement 5c**

We recommend considering restarting angiotensin-converting enzyme (ACE) inhibitor or angiotensin receptor blocker (ARB) therapy within the first 48 h postoperatively in patients who are haemodynamically stable without evidence of PO-AKI. This decision should be individualized and context-specific **(grade C evidence; strong recommendation)**.

**Consensus Statement 5d**

We recommend that enhanced recovery after surgery (ERAS) pathways (for example, use of NSAIDs and avoiding markedly positive fluid balance) are individualized based on the baseline risk of PO-AKI. The impact of ERAS pathways on the incidence of PO-AKI is unclear owing to the potential for both beneficial and harmful effects **(grade C evidence; strong recommendation)**.

## Treatment of postoperative AKI

As a general principal, treatment of AKI should be initiated as early as possible, including in patients with suspected AKI or who are at a substantially increased risk of AKI. Management of PO-AKI involves specific approaches, for example, avoidance of nephrotoxins, as well as general management principles that are common to all postoperative complications, such as haemodynamic optimization. Similarly, treatment of PO-AKI shares many features with the treatment of AKI in other settings (Table 1). Treatment goals include reducing kidney injury and complications related to decreased kidney function.

**Table 1:** Adaptation of the KDIGO guidelines for treatment of AKI to the postoperative setting

ADQI–POQI recommendations^a^	KDIGO strength of recommendation	KDIGO grade of evidence
In the absence of haemorrhagic shock, we suggest using a balanced and buffered isotonic crystalloid (e.g. Ringer’s lactate) rather than colloids (albumin or starches) as initial management for expansion of intravascular volume in patients with PO-AKI	Strong	B
We recommend the use of vasopressors in conjunction with fluids in patients with vasomotor shock with PO-AKI	Strong	D
We suggest using protocol-based management of haemodynamic and oxygenation parameters to treat patients with PO-AKI and to prevent worsening of AKI in high-risk patients in the perioperative setting	Strong	D
We suggest insulin therapy targeting plasma glucose <180 mg/dl (10 mmol) in patients with PO-AKI	Weak	Not graded
We suggest not using diuretics to treat AKI, except in the management of volume overload	Strong	A
We recommend not using low-dose dopamine fenoldopam, atrial natriuretic peptide or recombinant human IGF1 to treat AKI	Strong	A
We recommend not using nephrotoxic drugs in patients with PO-AKI unless no suitable, less nephrotoxic alternatives are available or the benefits outweigh the risks	Strong	A

ADQI, Acute Disease Quality Initiative; AKI, acute kidney injury; IGF1, insulin-like growth factor I. KDIGO, Kidney Disease Improving Global Outcomes; PO-AKI, postoperative AKI; POQI, PeriOperative Quality Initiative. ^a^Adapted from the treatment recommendations in the 2012 KDIGO clinical practice guideline for AKI^54^.

A full KHA should be performed, including drug history, which should focus on potential nephrotoxins, including antibiotics and radiocontrast agents. In addition to classical evaluation techniques, a number of biomarkers of cellular damage and functional change are available for the early diagnosis, risk assessment and prognosis of AKI^90–95^. Nephrology or urology consultation should be sought if intrinsic causes of AKI or obstruction are suspected based on the KHA results. The frequency and duration of monitoring should be individualized based on patient risk, exposure and clinical course (Box 6).

Box 6 Treatment of PO-AKI **Consensus Statement 6a**

We recommend that the underlying causes of postoperative acute kidney injury (PO-AKI) should be determined and treated whenever possible **(ungraded; strong recommendation)**.

**Consensus Statement 6b**

In patients with PO-AKI, we recommend that criteria for implementation of kidney replacement therapy (KRT) should be based on directly anticipated or observed life-threatening AKI-related complications **(grade D evidence; strong recommendation)**.

**Consensus Statement 6c**

We suggest that AKI phenotype is taken into account when deciding what type of treatment to implement **(ungraded; weak recommendation)**.

### The KDIGO bundle

The treatment and prevention bundle outlined in the 2012 KDIGO clinical practice guideline for AKI consists of supportive measures, including volume management, maintenance of adequate blood pressure and judicious avoidance of nephrotoxins. Two RCTs demonstrated that implementation of this bundle in high-risk patients identified by biomarkers after surgery significantly reduced the occurrence of PO-AKI, but were not powered to demonstrate differences in longer-term patient outcomes^96,97^. However, observational studies have shown that the severity of AKI correlates with short-term^98–101^ and longer-term^102^ adverse events, suggesting that interventions that reduce the severity of AKI could improve these outcomes.

### Kidney replacement therapy

Kidney replacement therapy (KRT) may be required in patients with severe AKI who develop medically refractory fluid and/or metabolic complications. In the absence of conventional indications, the best timing for initiating KRT in PO-AKI is uncertain. Potential rationales for initiating KRT during PO-AKI are avoidance of fluid accumulation, maintenance of acid-base and electrolyte control, provision of space to accommodate nutritional and medication needs, and abrogation of the interaction between the kidney and other organs. Theoretically, early initiation of KRT could facilitate better fluid, electrolyte and acid–base homeostasis. However, KRT is associated with increased risk of complications, healthcare costs and clinical workload.

The 2012 KDIGO guidelines recommend starting KRT “when life threatening changes in fluid, electrolyte, and acid-base balance exist” and considering the “broader clinical context, the presence of conditions that can be modified with [KRT], and trends of laboratory tests — rather than single BUN and creatinine thresholds alone — when making the decision to start [KRT]”. Contradictory trial results^103,104^ have stimulated debate regarding the best timing for KRT in patients with established AKI and differences in study design, patient populations, definitions of early and late RRT initiation, and inclusion criteria make head-to-head comparisons difficult.

Following our consensus conference, the results of the STARRT-AKI study provided high-quality evidence that early commencement of KRT based on AKI criteria in the absence of conventional indications does not improve survival, may needlessly expose some patients to KRT and results in a higher risk of KRT dependence at 90 days compared with a conservative strategy for starting KRT^105^. Importantly, STARRT-AKI included a large cohort of patients with PO-AKI (*n* = 965) and showed no evidence of a benefit of early commencement of KRT in this subgroup (odds ratio for mortality with earlier KRT: 1.20; 95% CI, 0.91–1.59). Thus, decisions regarding timing of KRT initiation remain a complex issue that should be individualized based on the clinical state of the patient, in particular imminently anticipated or observed life-threatening complications of AKI.

### AKI subtypes

AKI is a heterogeneous condition consisting of distinct endotypes and phenotypes based on its aetiology, prognosis and molecular pathways. Different subtypes might require and benefit from different therapeutic strategies. However, current AKI definitions based on serum creatinine and urine output provide no information on AKI subtypes. Studies using various damage and functional AKI biomarkers suggest that these new tools might overcome the limitations of current AKI definitions to improve AKI phenotyping^106^. Until these strategies are better embedded in clinical practice, the potential benefits of individualized therapy for AKI subtypes cannot be clearly established.

### Research recommendations

We recommend further trials to assess the optimal choice and volume of fluids for intravascular expansion and vasopressor therapy in patients with PO-AKI as well as the use of protocol-based management of haemodynamic and oxygenation parameters to prevent worsening of PO-AKI. Investigation of individual components of the KDIGO guideline to identify those that are the most effective in treating PO-AKI is important given that these were largely assembled based on expert opinion. Better understanding of AKI phenotypes is required to enable further trials to assess the response to implementation of the KDIGO bundle and other treatment options according to different AKI endotypes and phenotypes.

## Outcomes of postoperative AKI and AKD

**Fig. 4:**
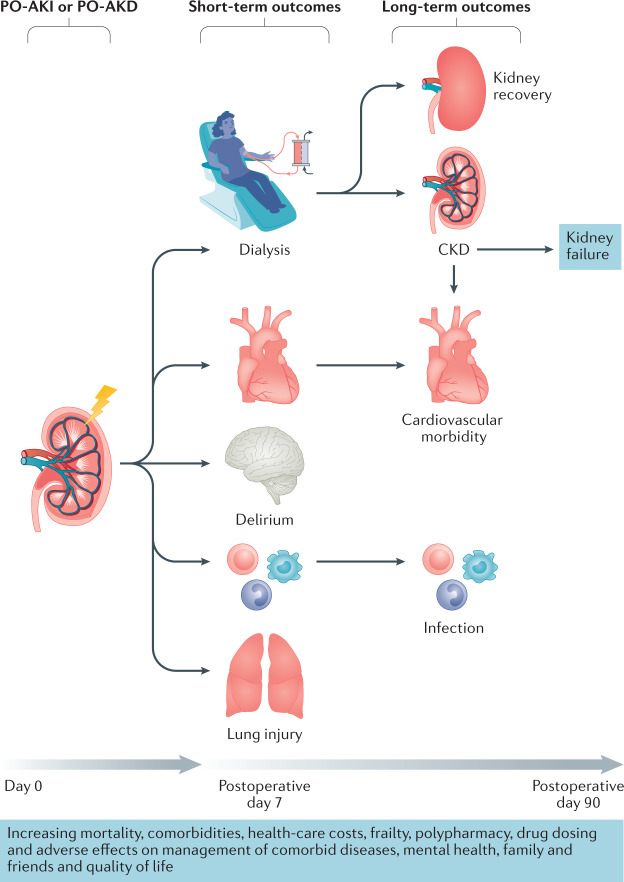
Outcomes of PO-AKI and PO-AKD. Postoperative acute kidney injury (PO-AKI) is associated with an increased risk of short-term adverse outcomes, including need for dialysis, cardiovascular events, lung injury, delirium and infection. These adverse effects can in turn lead to increased long-term morbidity and mortality. Adapted from Acute Disease Quality Initiative 24, www.ADQI.org, CC BY 2.0 (https://creativecommons.org/licenses/by/2.0/).

Regardless of the contributing mechanisms, PO-AKI has repeatedly been shown to be associated with a high risk of complications, including short-term and long-term mortality, high hospital costs and resource utilization^6,29,31,39,40,107–115^ (Fig. 4). Data from patients admitted to an ICU for at least 24 h following major surgery show that those who experienced PO-AKI had persistently worse survival over 10 years, even if kidney function recovered completely^6^. By contrast, a retrospective analysis of patients undergoing repair of ruptured abdominal aneurysm, a very high risk of acute exposure, suggested long-term survival was not significantly different between those with and without AKI, perhaps as a result of the overwhelming influence of immediate surgical outcome in this setting^31^.

Patients with PO-AKI or AKD are at an increased risk of recurrent episodes of AKI and progressive deterioration of kidney function, including CKD and dialysis dependency^39^. In patients undergoing major surgery, the incidence of kidney failure at 1 year was 0.94% among those who experienced AKI versus 0.05% in those who did not experience AKI^39^. Furthermore in a competing risk model accounting for death, adjusted progression to kidney failure during 10 years of follow-up was 0.4%, 2.3%, 7.3% and 15.7% for patients with no kidney disease, AKI with no CKD, CKD with no AKI and AKI with CKD, respectively (*P* < 0.001)^39^.

Other postoperative complications, including infection, prolonged mechanical ventilation, tracheostomy and cardiovascular events are also more common in patients who develop AKI after surgery than in those who do not^108^. Among 1,200 patients who underwent non-cardiac and non-vascular surgery, of whom 6.7% met the RIFLE criteria for AKI (2), the risk of non-cardiovascular complications (such as pneumonia and stroke) was similar in those with and without AKI over the first 2 weeks after surgery, but thereafter, patients with AKI experienced a significantly higher rate of cardiovascular events, including acute coronary syndrome, acute heart failure and arrhythmias^107^. Similarly, AKI after abdominal surgery has been associated with an increased risk of in-hospital complications, including perioperative acute myocardial infarction, pneumonia and sepsis (*P* < 0.001)^29^.

Unsurprisingly, the development of PO-AKI is associated with substantial increases in hospital costs and resource utilization, as well as longer stays in the ICU and in hospital^29,116,117^. Furthermore, the risks of 30-day re-admission and discharge to a nursing facility or rehabilitation centre are increased^31^. PO-AKI has also been associated with important, but difficult to quantify adverse outcomes, including reduced quality of life and perceived well-being^118^ as well as with increased risk of malignancy, possibly due to interactions with the immune system^119^.

### Postoperative monitoring and management

Given the increased risk of short- and long-term adverse outcomes following PO-AKI, appropriate monitoring and follow up of this cohort is a potential approach to improving patient outcomes. Although follow-up schemas for all-cause AKI have been proposed, the optimal timing of follow-up after a postoperative episode of AKI or AKD is unknown^58,120^ and limited evidence is available to support any specific monitoring paradigm. However, adverse outcomes are increased in patients with prolonged, more severe AKI, and in those whose kidney function does not recover to baseline levels, suggesting that use of these factors to inform the initial frequency and nature of follow-up would be a reasonable approach^121,122^.

**Fig. 5:**
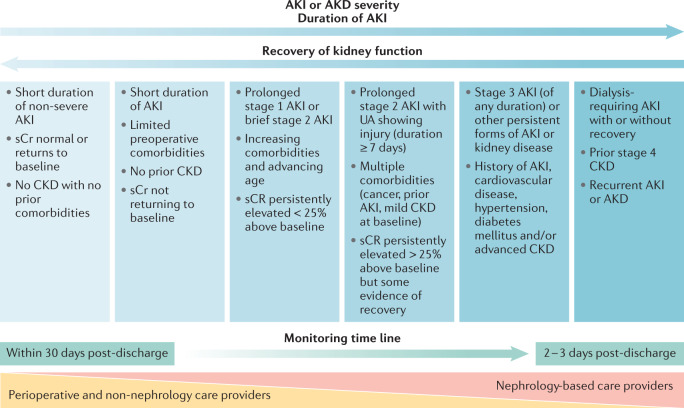
Potential monitoring approach for patients who experience PO-AKI or PO-AKD. Limited data are available to inform the timing and nature of monitoring for patients who experience postoperative acute kidney injury (PO-AKI) or postoperative acute kidney disease (PO-AKD). We suggest that these patients should have their kidney function checked within 1 month of hospital discharge to confirm the extent of recovery or progression of kidney disease. Those with persistent kidney dysfunction at 90 days should be formally assessed for the development or progression of chronic kidney disease (CKD). The degree of nephrology involvement in follow-up monitoring should increase with the duration and severity of AKI or AKD commensurate with the risk of developing CKD. Patients with less severe AKI or AKD can be monitored in primary care or by the base specialist and referred for nephrology care if needed according to CKD guidelines. Future research is needed to clarify the optimal timings and methods to provide post-AKI or AKD care. Adapted from Acute Disease Quality Initiative 24, www.ADQI.org, CC BY 2.0 (https://creativecommons.org/licenses/by/2.0/).

Monitoring pathways should be developed in conjunction with nephrologists, but may be directly provided by perioperative physicians, primary care physicians or other specialists (Fig. 5). As a minimum, we recommend that all postoperative patients with AKI or AKD have a KHA within 30 days of hospital discharge. To facilitate this assessment, accurate information transfer from the hospital team to the primary care team and the patient or care giver is essential. At the very least, a post PO-AKI KHA should consist of checking glomerular filtration (e.g. serum creatinine levels) as well as other persistent signs of kidney injury (e.g. albuminuria). Screening for albuminuria 3 months after the development of AKI has been shown to identify those patients who are at a greater risk of CKD progression^123^. When available, further work-up and monitoring of kidney function with other diagnostics may be appropriate. Changes in management based on this monitoring should be communicated with the patient as well as with other treating physicians.

In survivors of PO-AKI or AKD, the key priorities of long-term management are to facilitate kidney recovery, prevent progressive CKD and mitigate long-term comorbidities, including cardiovascular risk. However, whether any effective strategies beyond supportive CKD care exist is unknown. ADQI recommends that a post-AKI or AKD care bundle should include assessment of kidney function, advocacy and education, review and adjustment of medications, blood pressure management and a sick day protocol^58^. A multi-disciplinary follow-up approach (involving nephrology, primary care, pharmacy, a dietician, a social worker and non-nephrology subspecialists) combined with patient education is recommended but has not been formally studied. Undoubtedly, drug selection, dosing and monitoring are important and should be guided by personalized clinical decision making and regular assessment of kidney function. Both the use of nephrotoxic drugs and the inappropriate exclusion of potentially beneficial medications (for example, ACE inhibitors or antidiabetic medications) need to be avoided^124,125^. In patients with AKD, in particular, a substantial risk exists of potential therapeutic failure caused by underdosing or avoidance of effective cardiovascular drugs. Close collaboration between the relevant clinical teams is paramount and future research efforts should clarify the ideal timing and method for providing post-AKI and AKD care (Box 7).

Box 7 Outcomes of PO-AKI and PO-AKD **Consensus statement 7a**

The incidence of kidney and non-kidney adverse events is increased following postoperative acute kidney injury (PO-AKI) or postoperative acute kidney disease (PO-AKD). In particular, patients with surgery-associated AKI or AKD are at an increased risk of new or worsening chronic kidney disease (CKD), which is associated with increased long-term mortality **(grade B evidence)**.

**Consensus Statement 7b**

The prevention of new or worsening CKD following an episode PO-AKI or PO-AKD is a public health priority. We recommend that all patients undergo at least one kidney health assessment once the acute phase of AKI or AKD is complete. This approach will require optimal communication and AKI education between health-care professionals and the patient or caregiver **(grade B evidence; strong recommendation)**.

### Research recommendations

A need exists for epidemiological research to describe the frequency, nature and severity of PO-AKI and PO-AKD progression to CKD. The effectiveness and cost-effectiveness of supplementing a simple KHA with enhanced kidney health care in the prevention of CKD progression after surgery-associated AKI or AKD should be evaluated in appropriately selected patients. Finally, research is needed to explore a possible common aetiology between surgery-associated AKI or AKD and other chronic organ injuries as the longer-term effects of AKI do not only involve the kidney.

## Conclusions

PO-AKI is a sentinel postoperative event that is strongly associated with both short-term surgical complications and long-term adverse outcomes. In this Consensus Statement, we provide an overview of PO-AKI and management. However, PO-AKI is a heterogeneous syndrome with a variable clinical course. In many areas the strength of evidence is poor and accordingly our recommendations are weak. We provide research recommendations for these areas to enable greater clarity to be achieved in the future. Overall, we believe that an integrated approach to PO-AKI management requires serial evaluations of AKI risk, diagnosis, progress and outcomes throughout the perioperative period, which can be constructed as a series of focused KHAs.

## Supplementary information

Supplementary Information
