# Supplementary material for: Postoperative acute kidney injury in adult non-cardiac surgery: joint consensus report of the Acute Disease Quality Initiative and PeriOperative Quality Initiative
Source: Nat Rev Nephrol. 2021 May 11;17(9):605–18. doi: 10.1038/s41581-021-00418-2 (PMC8367817; doi:10.1038/s41581-021-00418-2)
Supplement: Supplementary file 1 — Supplementary Information [file 41581_2021_418_MOESM1_ESM.pdf]

nature portfolio

https://doi.org/10.1038/s41581-021-00418-2

Supplementary information

Postoperative acute kidney injury in adult non-cardiac surgery: joint consensus report of the Acute Disease Quality Initiative and PeriOperative Quality Initiative

In the format provided by the authors and unedited

Supplementary information

ADQI-24 and POQI-7 Faculty

Organizers/Chairs
John Prowle, QMUL, UK (Local Organiser)
John A. Kellum Pittsburgh, USA
Andy Shaw, University of Alberta, Canada
Lui Forni, Surrey University, UK
Claudio Ronco, University of Padua, Italy

Group 1 (Epidemiology and Pathophysiology)
Morgan Grams (Chair), Johns Hopkins University, USA
Mike Grocott (Co-Chair), Southampton University, UK
Raquel Bartz, Duke University, USA
Azra Bihorac, University of Florida, USA*
Lui Forni, Surrey University, UK

Group 2 (Definitions)
David McIlroy (Chair), Vanderbilt University, USA
Mitra Nadim (Co-Chair), USC, USA
Pat Murray, University College Dublin, Ireland
Ravi Mehta, USCD, USA
Andy Shaw, University of Alberta, Canada

Group 3 (Prevention)
Mark Edwards (Chair), Southampton, UK
Kathleen Liu (Co-Chair), UCSF, USA Tim Miller, Duke University, USA
Samira Bell, Dundee University, Scotland
Sean Bagshaw, University of Alberta, Canada
Michael Joannidis Univ Innsbruuck

Group 4 (Treatment)
Alex Zarbock (Chair), University of Munster, Germany
Michelle Chew (Co-Chair), Linkoping, Sweden
Denny Levett, Southampton University, UK
Monty Mythen, University College London, UK
Thomas Rimmele, Lyon, France
Chuck Hobson, University of Florida, USA*

Group 5 (Outcomes)
Marlies Ostermann (Chair), King's College UK
Max Bell (Co-Chair), Karolinska University, Sweden
Rupert Pearse, QMUL, UK
TJ Gan, Stony Brook University, USA
Jay Koyner, University of Chicago, USA
John Prowle, QMUL, UK

*Participated in pre-and post-conference activities only

Supplementary table 1. Incidence and risk factors for AKI after non-cardiac and non-vascular surgery

Study	Type of surgery	Study design	No. of Patients	AKI definition	AKI incidence	Risk factors for AKI
Long (2016)[29]	Open and laparoscopic abdominal surgery	Retrospective cohort	3,902	KDIGO	6.8%	Female gender, hypertension, CKD, ASA IV or V, reoperation
O'Connor (2016)[4]	Major abdominal surgery	Systematic review	Pooled 82,514	RIFLE, AKIN, KDIGO	13.4%	NA
Grams (2016)[31]	Major surgery	Retrospective cohort	161,185	KDIGO	11.8%	Older age, African American race, hypertension, diabetes, lower eGFR
Ishikawa (2012)[127]	Lung resection	Retrospective cohort	1,129	AKIN	5.9%	Hypertension, peripheral vascular disease, hetastarch use, lower eGFR, open procedure, prior use of ARB
Kim (2013)[128]	Gastric cancer surgery	Retrospective cohort	4,718	KDIGO	14.4%	Male gender, hypertension, COPD, hypoalbuminia, diuretics, vasopressors, contrast agents, PRBCs
Bravi (2019)[116]	Partial nephrectomy	Retrospective cohort	1,893	RIFLE	20.4%	Male gender, older age, lower eGFR, diabetes, hypertension, open surgery
Bang (2018)[129]	Colorectal Surgery	Retrospective cohort	4,320	AKIN, RIFLE	9.6%, 5.8%	NA
Bihorac (2013)[25]	Major surgery	Retrospective cohort	27,841	RIFLE	37%	NA
Hobson (2015)[130]	Inpatient surgery	Retrospective cohort	50,314	RIFLE	39%	Older, male gender, African American
Walker (2016)[131]	Orthopedic surgeries, neck of femur repair	Retrospective cohort	11,004	KDIGO	11.2%, 13.8%	NA
Vaught (2015)[132]	Major inpatient gynaecologic surgery	Retrospective cohort	2,341	RIFLE	13%	Older age, malignant tumor, metastatic cancer, emergent surgery, weekend admission, congestive heart failure, chronic pulmonary disease
Ozrazgat-Baslanti (2017)[113]	Major inpatient surgery	Retrospective cohort	51,457	KDIGO	39%	NA
Abdullah (2015)[23]	Laparoscopic bariatric surgery	Retrospective cohort	1,230	KDIGO	2.9%	Lower eGFR, angiotensin-converting enzyme inhibitors and angiotensin II receptor blockers, higher body mass index
Bonavia (2019)[26]	Colorectal surgery	Retrospective cohort	1,020	KDIGO (sCr only, sCr or urine output)	6.5%, 57.4%	NA
Kim (2016)[133]	Total knee arthroplasty	Retrospective cohort	1,309	KDIGO	4.4%	Age, diabetes, uric acid, beta blocker use, diuretics
Rajan (2015)[30]	Partial nephrectomy	Retrospective cohort	1,955	AKIN	39.3%	Hypertension, open and laparoscopic nephrectomy (vs robotic nephrectomy), duration of surgery, longer ischaemia time, and warm ischaemia (vs. cold ischaemia)

*Search strategy: studies with at least 1,000 patients, unselected for post-surgical outcomes (i.e., excluded studies of patients admitted to the ICU after surgery, or surviving until discharge).
